# The multisystemic effects of oral appliance therapy for obstructive sleep apnea: A narrative review

**DOI:** 10.1097/MD.0000000000029400

**Published:** 2022-07-22

**Authors:** Hee Young Kim, Jung Hwan Jo, Jin Woo Chung, Ji Woon Park

**Affiliations:** a Department of Oral Medicine, Seoul National University Dental Hospital, Seoul, Republic of Korea; b Department of Oral Medicine and Oral Diagnosis, School of Dentistry, Seoul National University, Seoul, Republic of Korea; c Dental Research Institute, Seoul National University, Seoul, Republic of Korea.

**Keywords:** cardiovascular disease, endocrine disorder, mood disorder, neurocognitive dysfunction, obstructive sleep apnea, sleep related breathing disorders, snoring

## Abstract

Obstructive sleep apnea (OSA) is a chronic condition accompanied by repeated obstruction of the upper airway during sleep despite respiratory efforts, resulting in intermittent hypoxemia, altered sleep structure, and sympathetic activation. Previous studies have shown a significant association between OSA and general health issues such as cardiovascular diseases, endocrine disorders, neurocognitive function decline, and poor quality of life. Continuous positive airway pressure (CPAP) has been considered as the first line treatment for OSA. However, accumulating evidence supports the role of oral appliance (OA) therapy, including mandibular advancement devices, as an alternative option for snoring and OSA patients who do not comply with or refuse CPAP usage. Despite a generally favorable outcome of OA therapy for OSA related respiratory indices, studies focusing on the impact of systemic effects of OA therapy in OSA patients are relatively scarce compared with the extensive literature focusing on the systemic effects of CPAP. Therefore, this article aimed to provide an overview of the current evidence regarding the multisystemic effects of OA therapy for OSA.

## 1. Introduction

Obstructive sleep apnea (OSA) is the most prevalent type of sleep-related breathing disorders affecting 33.9% of men and 17.4% of women between 30 and 70 years of age.^[1,2]^ The prevalence of OSA is increasing worldwide regardless of age, gender, and its severity of OSA.^[2]^ OSA is characterized by repeated partial or total obstruction of the upper airway during sleep despite respiratory effort, resulting in intermittent hypoxemia, altered sleep structure, and sympathetic excitation.^[1]^ Excessive daytime sleepiness is a hallmark symptom and patients have an increased risk for motor vehicle accidents.^[3]^ Moreover, OSA is commonly known to increase the risk of cardiovascular diseases,^[4–6]^ metabolic morbidity,^[7,8]^ systemic inflammation,^[9]^ and neurocognitive impairment^[10,11]^ resulting in poorer quality of life (QoL).^[12]^

Continuous positive airway pressure (CPAP) therapy is generally considered the gold standard approach for the treatment of OSA. However, its clinical effectiveness is often reduced due to a low acceptance rate and suboptimal patient compliance.^[13]^ Oral appliance (OA) therapy, especially mandibular advancement devices (MAD) have emerged as a noninferior alternative treatment option for snoring and mild to moderate OSA in patients who refuse CPAP or are intolerant to it.^[14–16]^ Recently, studies have shown that OA therapy may be effective in severe OSA as well.^[17]^ In a recent systematic review, OSA was successfully treated with OA therapy in 92% of the included subjects, and 77% of the subjects were compliant with the use of MAD.^[18]^ Despite a generally favorable outcome and higher patient preference for OA therapy, studies focusing on its systemic impact are relatively scarce compared with the extensive literature investigating the systemic effect of CPAP therapy. Therefore, the objective of this article was to provide an overview of the current evidence regarding the overall multisystemic effects of OA therapy in OSA patients.

## 2. Methods

### 2.1. Search strategy

This article is a narrative review, which describes and discusses the current state of knowledge regarding the effect of OA therapy for OSA on various systemic conditions. An electronic literature search was conducted on EMBASE and Ovid MEDLINE on July 7, 2021, independently by 2 researchers, and articles published between March 1977 and July 2021 were included in the final analysis. The search combined keywords referring to sleep apnea syndrome (obstructive sleep apnea, sleep apnea, sleep hypopnea, sleep disordered breathing) and terms associated with OA therapy (dental appliance or oral appliance or intraoral appliance). Reference lists from relevant review articles were also manually searched, and articles were further chosen when meeting the inclusion criteria.

### 2.2. Study selection

Two reviewers independently screened the title, abstract, and full text successively and decided on the eligibility of the searched article. Disagreement in choice was resolved through discussion until consensus was reached. Papers meeting the following inclusion criteria were included: written in English language; assessed treatment effect of OA therapy in OSA patients on at least one health-related outcome such as cardiovascular effects, inflammatory biomarkers, glucose metabolism, cognitive function, and mood disorders. The exclusion criteria were: abstract publication and commentaries.

**Figure 1.**
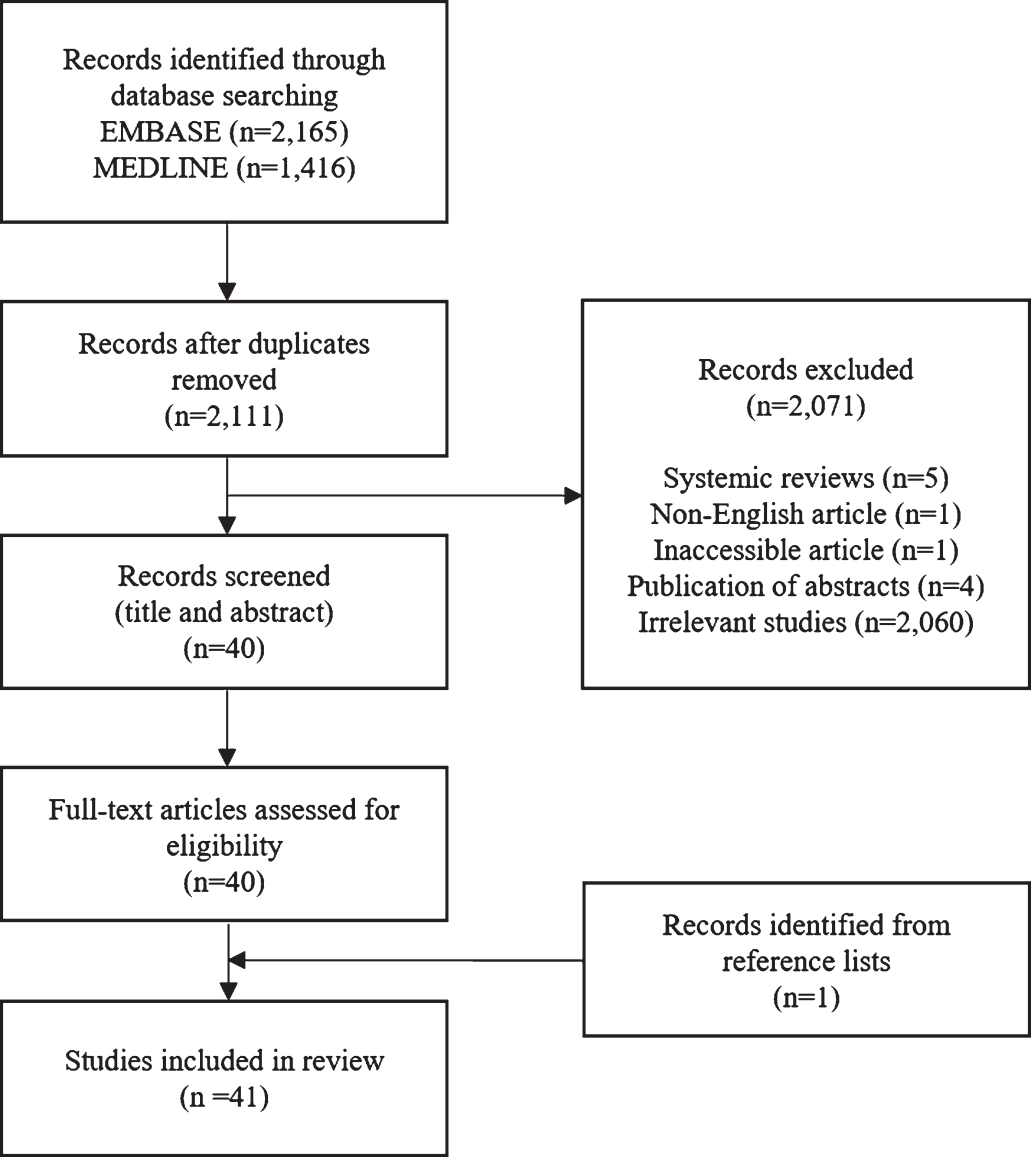
Flow chart of literature search and study selection process.

The literature search resulted in 3581 citations, of which 1470 were duplicates. After removing duplicates, a total of 2111 unique citations were included in the title and abstract screening process, which left 40 articles for full text review after the exclusion of 2071 citations. The excluded articles were systemic reviews (n = 5), non-English articles (n = 1), inaccessible article (n = 1), publication of abstract (n = 4), and irrelevant studies (n = 2060). After a complete inspection of the full texts, all 40 articles were included and 1 article from the reference list of a review paper was added. In total, 41 articles were selected for final analysis (Fig. 1).

The following data were extracted from the included articles: study design, study size, age, and sex of subjects included in the studies, clinical characteristics of the population for both OSA and systemic condition, and treatment outcomes. The data are summarized in Table 1. Since this was designed as a narrative review, the risk of bias of each individual study was not assessed. However, only studies with scientifically sound methodologies were selected through discussion between the 2 board certified clinicians with >4 and 16 years of clinical and academic experience, respectively, in the field of dental sleep medicine.

**Table 1:** Overview of the studies investigating systemic outcomes of oral appliance therapy for obstructive sleep apnea.

First author	Study design	Investigated systemic condition	Type of intervention	Study size (dropout)	Mean age, y	Malen (%)	Follow-up period	Baseline AHI/RDImean events/h	Post-treatment AHI/RDImean events/h	Outcome	Author's conclusion
Yoshida et al (2006).^[33]^ Japan	Observational	Cardiovascular - BP (mmHg)	MAD	161 (0)	54.3	121 (75)	2 mo	17.9	5.8	SBP 132.0–127.5 DBP 82.1–79.2	OA reduced daytime SBP and DBP.
Andrén et al (2009).^[34]^ Sweden	Observational	Cardiovascular - BP (mmHg)	MAD	29 (7)	57	18 (82)	3 mo 3 yr	16.1	4.1	SBP 154.9–139.4DBP 88.4–78.1	OA reduced daytime SBP and DBP.
Gauthier et al (2009).^[35]^ Canada	Single blind cross-over	Cardiovascular - BP (mmHg)	MAD - Type 1 - Type 2	16 (0)	47.9	11 (69)	2 × 3 mo	RDI 10.0	Type 1 7.8 Type 2 6.2	(Baseline/ Type 1/ Type 2)SBP 127.3/123.6/123.0DBP 91.0/85.0/84.6	OA showed a tendency to reduce SBP and DBP, but statistically significant only in DBP of Type 2.
Gauthier et al (2011).^[36]^ Canada	Observational	Cardiovascular - BP (mmHg)	MAD	14 (0)	51.9	10 (71)	40.9 mo	RDI 10.4	4.5	SBP 128.0–81.9 DBP 92.0–75.4	OA remained effective in improving BP for 2.5–4.5 years.
Otsuka et al (2006).^[37]^ Canada	Observational	Cardiovascular - BP (mmHg)	MAD	11 (0)	52.2	8 (73)	5.2 mo	RDI 24.7	6.1	20-h DBP 79.5–74.6 20-h MAP 95.9–91.2 sleep SBP 118.4–113.7 sleep DBP 71.6–67.2 sleep MAP 88.4–83.9	OA reduced 20-h DBP and MAP, sleep BP.
Gotsopoulos et al (2004).^[38]^ Australia	RCT cross-over	Cardiovascular - BP (mmHg) - HR (bpm)	MAD Inactive OA	67 (6)	48	53 (79)	2 × 4 wk	27	MAD 12 Inactive OA 24	(Baseline/Inactive OA/MAD)24-h DBP 77.7/78.0/76.4 awake SBP 131.1/129.7/126.7 awake DBP 80.6/80.5/77.3 awake HR 77/79/76	OA reduced mean 24 h DBP, and awake BP and HR compared with inactive OA.
Lam et al (2007).^[39]^ China	RCT parallel	Cardiovascular - BP (mmHg) Psychological	MAD CPAP Conservative measure (CM)	101 (0)	45.7	79 (78)	10 wk	MAD 20.9 CPAP 23.8 CM 19.3	MAD 10.6 CPAP 2.8 CM 20.5	morning DBP MAD 76.2–73.4 CPAP 77.0–71.8 CM 74.2–71.0 SF-36 (mental health) MAD 65.8–69.8 CPAP 66.8–71.8 CM 65.6–68.0	OA reduced morning DBP to the similar level as CPAP. None of the treatment modalities showed changes in the mental health domain of SF-36.
Andrén et al (2013).^[40]^ Sweden	RCT parallel	Cardiovascular - BP (mmHg)	MAD Inactive OA	72 (0)	58	57 (79)	3 mo	MAD 23 Inactive OA 24	MAD 7.6 Inactive OA 21.2	24-h SBP MAD 136.9–134.6 Inactive OA 139.3–138.8	Among the various BP parameters, OA reduced mean 24-h SBP the most compared with inactive OA in OSA patients with hypertension.
Trzepizur et al (2009).^[41]^ France	RCT cross-over	Cardiovascular - BP (mmHg) - Endothelial function	MAD CPAP	15 (3)	56*	11 (92)	2 × 2 mo	40^*^	MAD 14^*^CPAP 2^*^	(Baseline/MAD/CPAP)SBP 150/140/142DBP 61/63/64Ach-induced peak CVC (AU/mmHg)1.9/2.7/2.6	Both OA and CPAP improved endothelial function, but not BP.
Dal-Fabbro et al (2014).^[42]^ Brazil	RCT cross-over	Cardiovascular - BP (mmHg) - HRV	MAD CPAP Inactive OA	29 (0)	47	24 (83)	3 × 1 mo	42.3	MAD 26.7 CPAP 3.2 Inactive OA 48.7	(Baseline/MAD/CPAP/Inactive OA)24-h SBP 128.6/131.2/129.1/130.324-h DBP 80.6/80.6/79.5/80.3awake HR 82.5/81.0/80.0/81.9Total Power 17,536.2/15,576.9/14,608.6/19,108.8	BP parameters and HR did not change after any OSA treatment. Both CPAP and MAD significantly reduced total power, one of the HRV parameters, compared with inactive OA.
Sharples et al (2014).^[43]^ United Kingdom	RCT cross-over	Cardiovascular - BP (mmHg)	MAD - Type 1 - Type 2 - bespoke MAD (bMAD) Untreated	90 (16)	50.9	72 (80)	3 × 6 wk (4 wks for untreated period)	13.8	Type 1 10.8 Type 2 9.7 bMAD 9.5 Untreated 14.6	(Baseline/ Type 1/ Type 2/bMAD/Untreated)SBP 130.0/127.0/128.8/127.2/127.4DBP 80.4/79.0/79.9/79.5/79.2	None of the OA showed a significant difference in BP compared with the untreated group.
Yamamoto et al (2019).^[44]^ Japan	RCT cross-over	Cardiovascular - BP (mmHg) - Endothelial function	MAD CPAP	45 (5)	54.9	30 (75)	2 × 4 wk	28.6	MAD 8.9 CPAP 4.5	(Baseline/MAD/CPAP)24-hour SBP 125.8/124.1/125.324-hour DBP 79.8/78.8/79.0FMD 3.9/4.8/4.4	Neither CPAP nor OA significantly changed BP parameters and endothelial function.
Saletu et al (2007).^[48]^ Austria	Single blind placebo controlled case series	Cardiovascular - HR (bpm)	MAD Inactive OA	50 (0)	59.7	37 (74)	2–3 wk	16.8	MAD 7.7 Inactive OA 17.6	(Baseline/MAD/Inactive OA)Morning pulse rate 65.0/62.2/64.9	OA reduced morning pulse rate compared with inactive OA.
Galic et al (2016).^[49]^ Croatia	Prospective	Cardiovascular - HR (bpm) - Circulating biomarker Endocrine	MAD	18 (3)	51.2	14 (93)	3 mo 1 yr	22.9	3 mo 11.2 1 yr 9.7	HR 68.9–70.6 Fibrinogen (g/L) 3.4–3.0 Glucose (mmol/L) 5.3–4.9 Insulin (µU/mL) 14.1–10.9 HOMA-IR 3.3–2.4	OA did not change HR but reduced fibrinogen levels and improved glucose metabolism and insulin resistance.
Coruzzi et a (2006).^[53]^ Italy	Case-control	Cardiovascular - HRV	MAD Healthy control	20 (0)	45	11 (55)	3 mo	MAD 18.2 Control 2.6	MAD 4.2	(Control/pre-MAD/post-MAD) RRI (ms) 930/820/930 HF(ms^2^) 470/134/502	OSA patients had impaired cardiac autonomic function than healthy subjects, and OA improved the level of related parameters.
Kim et al (2020).^[54]^ Korea	Retrospective	Cardiovascular - HRV	MAD	58 (0)	53.1	51 (88)	3 mo	41.0	19.6	LFnu (ms^2^) 70.5–67.4 HFnu (ms^2^) 29.5–32.6	Improvements in frequency domain of HRV were seen only in patients successfully treated with OA.
Glos et al (2016).^[55]^ Germany	RCT cross-over	Cardiovascular - HRV	MAD CPAP	48 (8)	49.5	33 (83)	2 × 12 wk	28.5	MAD 13.7 CPAP 3.5	(Baseline/MAD/CPAP)LF (ms^2^) 26.6/32.4/37.4HF (ms^2^) 15.9/22.3/19.0LF/HF ratio 2.5/2.7/3.3	There were no differences between CPAP and OA with respect to HRV parameters after treatment.
Itzhaki et al (2007).^[63]^ Israel	Case-control	Cardiovascular - Endothelial function	MAD Untreated Healthy control	32 (4)	50.7	23 (72)	3 mo 1 yr	MAD 29.5 Untreated 31.0 Control 7.1	MAD 19.6 Untreated 29.2	Reactive hyperemia index Control 2.06MAD 1.77 to 2.0 Untreated 1.9 to 1.7	1-year of OA treatment significantly improved endothelial function to a similar extent of the healthy subjects.
Gagnadoux et al (2017).^[64]^ France	RCT parallel	Cardiovascular - Endothelial function	MAD Inactive OA	150 (21)	53.8	MAD (79) Inactive OA (93)	2 mo	MAD 40.0^*^ Inactive OA 47.0^*^	MAD 18.5^*^ Inactive OA 38.0^*^	Reactive hyperemia index MAD 2.13–2.10 Inactive OA 2.17–2.04	In severe OSA, OA had no effect on endothelial function despite the reduction in OSA severity.
Guimarães et al (2021).^[65]^ Brazil	RCT parallel	Cardiovascular - Endothelial function	MAD CPAP Untreated	79 (25)	46.9	43 (54)	6 mo 12 mo	MAD 9.3 CPAP 10.0 Untreated 9.3	MAD 3.8 CPAP 1.7 Untreated 11.6	Reactive hyperemia index MAD 2.0–2.3 CPAP 2.1–1.8Untreated 1.9–1.9	In mild OSA, 1-year of CPAP or OA did not improve endothelial function.
Lin et al (2015).^[66]^Taiwan	Case-control	Cardiovascular- Endothelial function	MADHealthy control	45 (0)	49.5	36 (80)	2 mo	MAD 31.6Control 3.2	MAD(success) 8.6MAD(fail) 24.7	FMDMAD(success) 5.9 to 10.5MAD(fail) data not definedControl data not defined	OSA patients had impaired endothelial function than healthy subjects. OA improved dilatation only in successfully treated patients.
Hoekema et al (2008).^[68]^ Netherlands	RCT parallel	Cardiovascular - Circulating biomarker	MAD CPAP	28 (3)	49.7	25 (89)	2–3 mo	MAD 31.7^*^ CPAP 54.8^*^	MAD 2.4^*^ CPAP 2.0^*^	NT-pro-BNP (pg/mL)^*^ MAD 52–22 CPAP 31–37	In moderate to severe OSA, OA significantly improved NT-pro-BNP levels.
Recoquillon et al (2019).^[69]^ France	RCT parallel	Cardiovascular - Circulating biomarker Endocrine	MAD Inactive OA	150 (41)	53.6	MAD (82) Inactive OA (94)	2 mo	MAD 40.0^*^ Inactive OA 44.5^*^	MAD 17.5^*^ Inactive OA 38.5^*^	NT-pro-BNP (pg/mL) MAD 296.8–252.5 Inactive OA 189.8–184.3TNF-a (pg/mL)MAD 6.7–6.4Inactive OA 8.1–6.7	In severe OSA, OA did not affect circulating biomarker levels (NT-pro-BNP, CRP, IL-6, TNF-a, and its receptors, adiponectin, leptin, and P-selectin) and metabolic parameters compared with inactive OA.
Halawani et al (2018).^[70]^ USA	Prospective	Cardiovascular - Circulating biomarker	MAD	22 (0)	66^*^	21 (96)	3 mo	21.35^*^	MAD(success) 7.95^*^ MAD(fail) 31.45^*^	Neutrophil-to-lymphocyte ratio^*^ MAD (success) 2.93–1.80 MAD fail) 2.64–2.5	OA significantly improved neutrophil-to-lymphocyte ratio values only in the optimally treated group.
Fernández et al (2018).^[71]^ Spain	Case-control	Cardiovascular - Circulating biomarker	MAD Untreated	40 (0)	55	24 (60)	6mo	MAD 28.7^*^ Untreated 24^*^	MAD 6.9^*^ Untreated 23.8^*^	IL-ß (pg/mL)^*^ MAD 0.9–0.4 Untreated 0.9–1.1 TNF-a (pg/mL)^*^ MAD 11.3–3.2 Untreated 13.2–12.2	In moderate to severe OSA, OA significantly improved inflammatory marker levels (IL-ß, TNF-a) compared with untreated patients.
Hedberg et al (2020).^[72]^ Sweden	RCT parallel	Cardiovascular - Circulating biomarker	MAD Inactive OA	72 (1)	59*	56 (79)	3 mo	MAD 19^*^ Inactive OA 19^*^	MAD 7^*^ Inactive OA 17^*^	IL-6 (ng/L)^*^MAD 1.00–1.02Inactive OA 1.04–0.98TNF--a (ng/L)^*^MAD 1.25–1.33Inactive OA 1.10–1.14	In OSA patients with hypertension, OA did not affect circulating inflammatory marker levels (white blood cells count, CRP, IL-6, IL-10, and TNF-a).
Baslas et al (2019).^[80]^ India	Observational	Endocrine	MAD	24 (0)	45.5	18 (75)	3 mo	Mild OSA 8.08 Moderate OSA 22.38 Severe OSA 38.09	Mild OSA 2.95 Moderate OSA 6.11 Severe OSA 18.59	HbA1c (%) Mild OSA 6.69–5.78 Moderate OSA 6.95–6.11 Severe OSA 6.94–6.89	OA significantly improved HbA1c levels in mild to moderate OSA patients, but not in severe OSA patients.
E Silva LO et al (2021).^[81]^ Brazil	RCT parallel	Endocrine	MAD CPAP Untreated	79 (25)	46.7	43 (54)	6 mo 1 yr	MAD 9.3 CPAP 10.0 Untreated 9.3	MAD 3.8 CPAP 1.7 Untreated 11.6	Total cholesterol (mg/dL) MAD 192.8–193.6 CPAP 189.3–173.4 Untreated 175.6–185.6 LDL cholesterol (mg/dL) MAD 116.6–118.4CPAP 112.8–94.5 Untreated 104.1–107.0	In mild OSA, only CPAP reduced total cholesterol and LDL cholesterol levels, and neither treatment improved glucose and insulin parameters.
Naismith et al (2005).^[90]^ Australia	RCT cross-over	Cognitive psychological	MAD Inactive OA	73 (0)	48.4	59 (81)	2 x 4 wk	26.9	MAD 12.2 Inactive OA 25.4	(Baseline/MAD/Inactive OA)Choice reaction time 0.664/0.643/0.662 BDI (Somatic items) 3.3/2.1/2.7	OA improved vigilance/psychomotor speed compared with inactive OA, and was associated with improvement in somatic items of BDI.
Tegelberg et al (2012).^[91]^ Sweden	Prospective	Cognitive	MAD	50 (7)	52	50 (100)	6 mo	40.9	18.0	WM test (Distraction task) 85.4–87.7 CPT (Correct counting) 53.1–62.7CPT (D’prime) 1.201–1.419 TMT (part A) 34.6–29.0	In moderate to severe OSA, OA improved attention/vigilance (WM, CPT) and motor speed (TMT).
Gupta et al (2017).^[92]^ India	Prospective	Cognitive	MAD	30 (0)	41	25 (83)	6 mo 1 yr 2 yr	22.00	6 mo 6.0 1yr 3.8 2 yr 3.53	PVT (response time): data no defined	OA showed marked improvement in response time of PVT, which measured speed, accuracy, and mental endurance.
Galic et al (2016).^[93]^ Croatia	Prospective	Cognitive	MADHealthy control	33 (3)	51.2	Data not defined	3 mo 1 yr	22.9	3mo 11.2 1yr 9.7	Simple arithmetic (CRD 11)- MinT(s), TTST(s)Control 2.29, 132.83Pre-MAD 2.64, 155.36Post-MAD 2.31, 137.09	OA significantly improved performance in the CRD series, indicating improvement in perceptive abilities, convergent thinking, and psychomotor reaction time.
Phillps et al (2013).^[94]^ Australia	RCT cross-over	Cognitive QoL	MAD CPAP	126 (18)	49.5	102 (81)	2 × 1 mo	25.6	MAD 11.1 CPAP 4.5	(Baseline/MAD/CPAP)AusEd driving (Mean Rt to DAT)1.05/0.98/0.97	In moderate to severe OSA, both OA and CPAP significantly improved the driving simulator performance and disease-specific QoL in a similar extent.
Barnes et al (2004).^[105]^ Australia	RCT cross-over	Psychological	MAD CPAP Placebo tablet	104 (24)	47	83 (80)	3 × 3 mo	21.3	MAD 14.0 CPAP 4.8 Placebo 20.3	(Baseline/MAD/CPAP/Placebo)BDI 9.2/6.9/6.7/7.7	All treatment modalities decreased BDI scores, and there was no intergroup difference, suggesting a placebo effect.
Blanco et al (2005).^[107]^ Spain	RCT parallel	Psychological	MAD Inactive OA	20 (5)	53.5	13 (87)	3 mo	MAD 33.8 Inactive OA 24.0	MAD 9.6 Inactive OA 11.7	SF-36 (Mental health domain) MAD 60.1–59.4 Inactive OA 52.0–56.0	OA did not improve all dimensions of SF-36 and showed no significant difference with inactive OA.
Petri et al (2008).^[108]^ Denmark	RCT parallel	Psychological QoL	MAD Inactive OA Untreated	93 (12)	49.6	66 (81)	4 wk	MAD 39.1 Inactive OA 32.6 Untreated 34.3	MAD 25.0 Inactive OA 31.7 Untreated 33.3	SF-36 (Mental health domain) MAD 71.0–76.4 Inactive OA 78.4–80.4 Untreated 79.6–79.0	There was no significant difference between the three groups in mental health domain of SF-36 after treatment.
Park et al (2021).^[115]^ Canada	Retrospective	Headache	MAD	13 (0)	49.9	4 (31)	5.7 yr	15.40	7.2	Headache frequency(days/3 month)6.00–1.50^*^Severest headache intensity (VAS)7.88–4.43	OA reduced headache frequency and presence of morning headache in OSA patients with headaches.
Marklund et al (2007).^[117]^ Sweden	Observational	Headache	MAD	260 (75)	52	198 (76)	5.4 yr	15	Data not defined	Morning headaches frequency1 (yearly or never) to 1^*^	OA reduced self-reported morning headaches in OSA patients.
Marklund et al (2015).^[118]^ Sweden	RCT parallel	Headache QoL	MAD inactive OA	96 (5)	51.9	62 (68)	4 mo	MAD 15.6 Inactive OA 15.3	MAD 6.7 Inactive OA 16.7	Headache present (%)MAD 84 to 71Inactive OA 77 to 70	Headache characteristics and all domains of the SF-36 were not differ between OA and inactive OA.
El-Solh et al (2017).^[121]^ USA	RCT cross-over	QoL	MAD CPAP	42 (7)	52.7	Data not defined	2 × 12 wk	34.7	MAD 26.3 CPAP 3.9	PTSD Checklist (PCL-M) Baseline data not definedMAD 6.22 CPAP 4.29	Both CPAP and OA improved PTSD severity and QoL in PTSD veterans, with no differences between the two treatment modalities.
Lin et al (2019).^[133]^ Taiwan	Case-control	Telomere length	MAD Healthy control	60 (0)	47.3	47 (78)	3mo	OSA 38.3 Control 3.9	MAD (success) 8.6 MAD (fail) 29.8	Telomere length 0.558–0.662 SIRT1 (pg/µg) 0.53–0.79	OA significantly increased leukocyte telomere length and SIRT1 protein level in OSA patients.

Ach = acetylcholine, AHI = apnea-hypopnea index, BDI = Beck Depression Inventory, BP = blood pressure, CPAP = continuous positive airway pressure, CPT = Continuous Performance Test, CRD = Complex Reactionmeter Drenovac, CRP = C-reactive protein, CVC = maximal cutaneous vascular conductance, DBP = diastolic blood pressure, FMD = flow-mediated dilatation, HF = high frequency, Hfnu = high-frequency power in normalized units, HOMA-IR = homeostatic model assessment-estimated insulin resistance, HR = heart rate, HRV = heart rate variability, IL = interleukin, LDL = low-density lipoprotein, LF = low frequency, Lfnu = low-frequency power in normalized units, MAD = mandibular advancement device, MAP = mean arterial pressure, NT-pro-BNP = N-terminal pro-brain-type natriuretic peptide, OA = oral appliance, PVT = Psychomotor vigilance test, PTSD = posttraumatic stress disorder, PCL-M = PTSD CheckList - Military Version, QoL = quality of life, RDI = respiratory disturbance index, RCT = randomized controlled trial, Rt to DAT = reaction time to divided attention task, RRI = R-R interval, SBP = systolic blood pressure, SF-36 = 36-Item Short Form Health Survey, SIRT1 = Sirtuin 1, TMT = Trail-Making Test; TNF = tumor necrosis factor, VAS = visual analog scale, WM test = Working memory test.

* Median value.

This narrative review is based on data from previous papers and does not need ethical approval since no human subjects were involved.

## 3. Discussions

### 3.1. Cardiovascular system

There is considerable evidence supporting an independent association between OSA and cardiovascular diseases.^[4,6]^ Sympathetic nervous system activation resulting from intermittent hypoxia, systemic inflammation,^[9,19]^ increased oxidative stress,^[20]^ and dysfunction of the vascular endothelium^[21]^ are considered the underlying mechanism. CPAP has been considered as the first line treatment approach for moderate to severe OSA and is known to prevent new-onset hypertension, cardiovascular and cerebrovascular diseases, eventually reducing all-cause mortality.^[5]^ In many studies, CPAP has been shown to improve hypertension,^[22]^ systemic inflammation,^[23]^ endothelial dysfunction,^[24]^ and cardiovascular morbidity and mortality.^[25]^ However, there are relatively limited data on the effect of OA therapy for OSA on cardiovascular outcomes, and most studies were focused on the effect of MAD on blood pressure.

#### 3.1.1. Blood pressure.

Several large epidemiological studies have supported the role of OSA as an independent risk factor for hypertension.^[26–28]^ The prevalence of OSA was significantly higher in the hypertensive group compared with the non-hypertensive group,^[29,30]^ especially in patients with refractory hypertension who were resistant to antihypertensive drug therapy.^[31]^ A meta-analysis of randomized controlled trials (RCT) found that CPAP reduced systolic blood pressure (SBP) and diastolic blood pressure (DBP) by 2.46 and 1.83 mmHg, respectively, in OSA patients.^[32]^ Studies regarding the effect of OA therapy on blood pressure (BP) are not sufficient and have shown conflicting results depending on study design, BP measurement method, or OSA severity of the included patients.

The results of several observational studies showed that MAD treatment in general improved BP from baseline. Yoshida^[33]^ and Andrén et al^[34]^ reported significant reductions in office SBP and DBP after 2-months and 3-years of MAD treatment, respectively. Gauthier et al^[35]^ compared 2 types of OAs, and both devices decreased SBP and DBP after 3-months, but the change was significant only in patients’ DBP with a certain type of OA. Meanwhile, in a follow-up study (mean usage duration of 40.9 ± 2.1 months) with the same participants, both SBP and DBP were significantly reduced from baseline.^[36]^ Otsuka et al^[37]^ used a 20-hour ambulatory blood pressure monitoring to monitor changes in BP and reported significant reductions in 20-hour DBP and mean arterial pressure, and in sleep SBP, DBP, and mean arterial pressure following MAD treatment. Awake BP decreased, but the change was not significant.

Similar results were seen in other controlled studies. Gotsopoulos et al^[38]^ compared 4-weeks of MAD with an inactive OA, and BP was assessed with 24-hour ambulatory blood pressure monitoring. Mean 24-hour DBP was significantly decreased with MAD compared with an inactive OA, but unlike Otsuka et al,^[37]^ there was no significant difference in sleep BP. Lam et al^[39]^ examined 101 mild to moderate OSA patients who received either CPAP, MAD, or sleep hygiene instructions for 10-weeks. Both MAD and CPAP significantly lowered morning DBP from baseline, and there was no difference in BP between the 2 groups. Andrén et al^[40]^ compared 3-months of MAD with an inactive OA in OSA patients with hypertension. Among BP parameters, mean 24-hour SBP decreased the most compared with inactive OA.

However, some found no significant changes in BP after OA therapy. Trzepizur et al^[41]^ reported that neither CPAP nor MAD for 2 months improved BP. In a study by Dal-Fabbro et al,^[42]^ patients with moderate to severe OSA were treated with CPAP, MAD, and an inactive OA for 1 month each. None of the treatments were associated with a significant change in BP parameters. There was a more evident DBP dipping pattern with MAD compared with CPAP. Sharples et al^[43]^ compared 3 types of MAD against no treatment, but none showed a significant difference in BP compared with the no treatment group after 4 weeks. Recently, Yamamoto et al^[44]^ also compared CPAP and MAD for 2 months respectively in moderate to severe OSA, but none of the BP parameters were significantly improved by either therapy.

Data from individual studies on the effect of MAD on BP are inconclusive, but systematic reviews and meta-analyses have reported modest reductions in BP by OA compared with baseline. In a meta-analysis by Iftikhar et al,^[45]^ SBP, DBP, and nocturnal DBP were significantly decreased by 2.7, 2.7, and 1.7 mmHg, respectively, after OA therapy in mild to moderate OSA patients. These effects were comparable to those previously reported with CPAP treatment.^[32]^ In a recent meta-analysis,^[46]^ MAD treatment reduced daytime SBP and DBP by 1.81 and 2.21 mmHg, respectively, from baseline, but the reduction in BP was less evident than in earlier meta-analyses. Unlike Iftikhar et al,^[22,45]^ which included many observational studies, the recent meta-analysis was performed based on RCTs. Also, the reduction in BP with OA was not statistically significant compared with control groups, as the weight loss of the control group may have affected BP. In another systematic review comparing the effect of CPAP and MAD on BP changes with controls, MAD was associated with significant reductions in SBP and DBP by 2.1 and 1.9 mmHg, respectively, and there was no significant difference in BP change occurring with CPAP and MAD.^[47]^

#### 3.1.2. Heart rate and heart rate variability.

##### 3.1.2.1. Heart rate.

Only a small number of studies have investigated the effects of OA therapy on heart rate (HR). In a study by Gotsopoulos et al,^[38]^ 4 weeks of MAD treatment significantly reduced awake HR compared with an inactive OA, but no significant difference was observed in sleep HR between the groups. Saletu et al^[48]^ also reported a significant decrease in morning pulse rate after the night using MAD compared with the night with inactive OA. There was no significant change in the evening pulse rate in this study. In contrast, in a study by Dal-Fabbro et al,^[42]^ 29 patients received MAD, CPAP, and inactive OA treatment for 1 month each, but there was no change in HR with any treatment. Galic et al^[49]^ also found no significant difference in HR before and after 1 year of MAD therapy. In a recent meta-analysis regarding the cardiovascular effects of OA therapy for OSA, the 2 above studies were included, and the pooled data showed a significant reduction in daytime HR.^[46]^

##### 3.1.2.2. Heart rate variability.

Heart rate variability (HRV), which reflects sympathetic and parasympathetic modulation, is defined as the variation in the time interval between adjacent heartbeats. HRV is measured based on electrocardiography signals.^[50]^ OSA patients showed an increased risk of developing HRV aberrations,^[51]^ and a correlation between apnea-hypopnea index (AHI) and HRV parameters has been observed.^[52]^

Coruzzi et al^[53]^ investigated the effect of MAD on daytime HRV in 10 OSA patients and 10 healthy subjects. At baseline, normal-to-normal interval and high frequency was significantly decreased in the OSA group compared with the control group. After 3 months of MAD treatment, these parameters were significantly improved, and differences between the two groups were no more significant. Kim et al^[54]^ also evaluated nocturnal HRV in 58 OSA patients and found significant positive changes following 3 months of MAD therapy. In particular, improvements in frequency domain variables were only significant in the MAD response group (defined as a >50% decrease in baseline AHI and <20/h post-treatment). In controlled studies, Dal-Fabbro et al^[42]^ compared 1-month of CPAP, MAD, and inactive OA. Both CPAP and MAD significantly reduced the total power of HRV compared with inactive OA. Glos et al^[55]^ also found no differences between MAD and CPAP for daytime cardiac autonomic function changes, although the reduction in AHI was superior in CPAP than MAD.

Alteration in HR and HRV have been reported in OSA patients^[51,56]^ and was associated with increased cardiovascular mortality.^[57–59]^ Although the beneficial effects of OA therapy in OSA patients on both variables have been observed in some studies, the results are inconclusive due to the limited amount of data and heterogeneity, calling for further studies on the issue.

#### 3.1.3. Endothelial function.

Endothelial dysfunction occurs early in the development of atherosclerosis and plays an important role in the pathogenesis of coronary artery disease. Various techniques, both invasive and non-invasive, are used to evaluate endothelial function in patients with OSA, and several studies have suggested its impairment,^[60]^ increased arterial stiffness,^[61]^ and early signs of atherosclerosis^[62]^ in OSA patients. Although treatment of OSA may reduce overall cardiovascular risk by improving vascular function, a limited number of studies have investigated the effect of OA therapy on the endothelial function itself.

Some studies have evaluated endothelial function with the reactive hyperemia index measured by peripheral arterial tonometry. In the study by Itzhaki et al,^[63]^ endothelial function improved after 3 months and 1 year of MAD treatment, and post-treatment status was not different from those without OSA. In contrast, Gagnadoux et al^[64]^ compared MAD with an inactive OA, where MAD therapy significantly improved AHI and related symptoms but did not affect endothelial function. Similarly, Guimarães et al^[65]^ found no significant changes in endothelial function despite effective OSA treatment with MAD or CPAP for 6 and 12 months.

Trzepizur et al^[41]^ assessed microvascular endothelial function based on laser Doppler flowmetry. Acetylcholine-induced vasodilation was significantly impaired in patients with OSA compared with controls. Both 2 months of CPAP and MAD therapy significantly increased acetylcholine-induced vasodilation and was correlated with improved nocturnal oxygenation. Lin et al^[66]^ used the endothelium-dependent flow-mediated dilation method and found dilatation was lower in OSA patients. After 2 months, significant improvement in dilatation was seen only in patients who were successfully treated with MAD (defined as post-treatment AHI <5% or >50% decrease in AHI but post-treatment AHI >5). However, in an RCT by Yamamoto et al^[44]^ neither MAD nor CPAP significantly changed endothelial function measured by flow-mediated dilation. In a recent meta-analysis, Cammaroto et al^[67]^ analyzed the impact of various OSA treatments on endothelial function and found that the extent of improvement with MAD was significant and similar to that from other treatments such as CPAP, surgery, and pharmacological therapy.

Most included studies showed that OA therapy could generally improve endothelial function, but their significance was limited due to the small study size. Meanwhile, the effects of CPAP on endothelial function are well established, and a recent systemic review demonstrated that CPAP significantly improved endothelial function to a clinically significant extent.^[24]^

#### 3.1.4. Circulating cardiovascular biomarkers.

Few studies investigated the effect of MAD on circulating cardiovascular biomarkers, and the outcome parameters used varied from study to study, with the results being highly heterogeneous.

##### 3.1.4.1. N-terminal pro-brain-type natriuretic peptide (NT-pro-BNP).

Hoekema et al^[68]^ investigated NT-pro-BNP levels in 28 patients with moderate to severe OSA, who were treated with either MAD or CPAP for 2 to 3 months. The median NT-pro-BNP levels improved significantly following MAD treatment (52–22 pg/mL) compared with CPAP therapy (31–37 pg/mL), but the authors stated that such results should be cautiously interpreted as baseline NT-pro-BNP levels were higher in the MAD group. Meanwhile, in a study by Recoquillon et al^[69]^ comparing MAD with a sham device, there was no difference in NT-pro-BNT levels between the 2 groups after treatment.

##### 3.1.4.2. Inflammatory biomarkers.

Al-Halawani et al^[70]^ reported a significant change in the neutrophil–lymphocyte ratio, a hematologic index of systemic inflammation, after 3-months of MAD treatment. In subgroup analysis, only the optimally treated group showed a significant decrease.

Galic et al^[49]^ prospectively investigated mild to moderate OSA patients and found a significant decrease in plasma fibrinogen level after 1-year of MAD therapy. Fernández et al^[71]^ compared moderate to severe OSA patients who received MAD treatment with those who refused any treatment. After 6 months, interleukin (IL)-1β and tumor necrosis factor (TNF)-α levels were significantly decreased in the MAD group compared with the untreated group. However, recent controlled studies using sham devices failed to show significant differences in inflammatory marker levels after OA treatment. In a study by Recoquillon et al,^[69]^ no significant changes of any circulating biomarker levels (C-reactive protein [CRP], IL-6, TNF-α, and its receptors, adiponectin, leptin, and P-selectin) were found with MAD compared with the sham devices. Hedberg et al^[72]^ did not find any significant differences between MAD and inactive OA in any inflammatory marker levels (white blood cell count, high-sensitivity CRP, IL-6, IL-10, and TNF-α) after the 3-month treatment period. A treatment period of 2 to 3 months may be insufficient to have an impact on circulating biomarker levels. However, it is uncertain whether extended follow-up with OA therapy could have any meaningful effects given the findings of a previous RCT of 1 year CPAP treatment also failing to show any changes in the level of circulating inflammatory markers.^[73]^

### 3.2. Endocrine system

Metabolic syndrome is a common metabolic disease characterized by glucose intolerance, obesity, hypertension, and dyslipidemia^[74]^ which is known to affect 35% to 40% of the adult population in the United States.^[75]^ Metabolic syndrome and OSA have several overlapping risk factors, including middle age, sedentary behavior, poor diet, and genetics.^[76]^ Results of epidemiological studies indicate that individuals with OSA are 6 to 9 times more likely to develop metabolic syndrome than the general population.^[77,78]^ Additionally, the prevalence of type 2 diabetes in individuals with OSA is estimated at 15% to 30% with a higher prevalence in severe OSA.^[79]^ Intermittent hypoxemia and sleep fragmentation, both cardinal features of OSA, are likely to be a component of the causal pathway leading to metabolic dysfunction.^[76,79]^ Despite its clinical importance, studies analyzing the effects of OA therapy on metabolic outcomes are relatively rare.

Galic et al^[49]^ investigated the effect of MAD on glucose metabolism in mild to moderate OSA patients. A significant decrease in AHI was observed after 1-year of MAD therapy along with a significant decrease in metabolic parameters, including fasting plasma glucose and insulin, and homeostatic model assessment-estimated insulin resistance. Baslas et al^[80]^ examined HbA1c levels before and after 3-months of MAD treatment in 24 patients with OSA and type 2 diabetes. Significant improvement in HbA1c level was seen only in mild and moderate OSA patients and not for severe patients.

In contrast, a study by Recoquillon et al^[69]^ compared 2 months of MAD therapy with an inactive device in severe OSA patients. The results showed no effect on metabolic parameters despite high objective adherence to treatment and a significant reduction in OSA severity. The author stated that the 2-month intervention period, which was shorter compared with the 2 previously mentioned studies, may have influenced the results. However, the last 2 studies have limitations as they were uncontrolled studies. Similarly, Silva et al^[81]^ compared MAD with CPAP in mild OSA patients, but the total and low-density lipoprotein cholesterol levels were significantly improved only in the CPAP group, and glucose and insulin parameters did not show significant changes in either group. Despite the strong association between OSA and metabolic dysfunction, a systematic review of cardiometabolic biomarkers concluded that CPAP did not significantly improve glucose, lipid, and insulin resistance levels nor the percentage of patients with metabolic syndrome.^[82]^ Another recent review also reported that CPAP did not improve HbA1c levels in individuals with type 2 diabetes.^[76]^

Meanwhile, a recent RCT compared the effectiveness of combination therapy over CPAP or weight loss alone.^[83]^ After 6-months of treatment, CPAP alone did not improve any cardiovascular markers, whereas weight loss and combined intervention improved inflammation, insulin resistance, and serum triglyceride levels. Although visceral fat is a key predictor of both OSA and metabolic syndrome, CPAP treatment alone cannot reduce visceral fat in patients with OSA, suggesting the need for additional weight loss.^[84]^ Taken together, improvements in AHI and sleep efficiency without weight loss are not sufficient to successfully modify cardiometabolic profiles, and there is a need to provide various treatment modalities simultaneously for OSA patients.

### 3.3. Cognitive function

OSA patients have an increased risk of cognitive impairment than those without OSA.^[11]^ A recent meta-analysis showed that OSA patients demonstrate decreased attention, episodic and working memory, and executive function while verbal function was preserved and mixed results were obtained for psychomotor speed.^[85]^ Intermittent hypoxia and the resulting sleep fragmentation appear to be partially associated with impaired cognitive function in the OSA population.^[86]^ Several studies have shown that CPAP treatment could improve the cognitive impairment associated with OSA. In particular, attention, executive function, and episodic memory were partially improved after CPAP treatment, whereas psychomotor speed and fine coordination were not.^[87–89]^ However, unlike CPAP, few studies have evaluated the effect of OA therapy on cognitive changes in OSA.

In an RCT, Naismith et al^[90]^ evaluated the effect of MAD treatment on neuropsychological function and mood state in 73 OSA patients. Both MAD and an inactive device were applied for 4 weeks. MAD treatment resulted in significant improvement within the neuropsychological domains of vigilance/psychomotor speed. Tegelberg et al^[91]^ also examined 50 male patients with moderate to severe OSA, and there was a significant improvement in attention/vigilance and motor speed domain after 6 months of MAD treatment. Similarly, a study by Gupta et al^[92]^ found a significant decrease in response time on psychomotor vigilance tests indicating improvements in speed, accuracy, and mental endurance after MAD treatment. In a study by Galic et al^[93]^ on 15 mild to moderate OSA patients, their general ability to perform in problem situations at baseline showed significant improvement after 1 year of MAD treatment in perceptive abilities, convergent thinking, and psychomotor reaction time. Phillips et al^[94]^ compared the effects of MAD and CPAP on driving simulator operation ability in moderate to severe OSA patients. Although the reduction in AHI was superior with CPAP, both treatments significantly improved operation ability from baseline. The higher adherence rate to MAD treatment may be relevant to such results.

Overall, OA therapy in OSA patients resulted in marked improvement in attention/vigilance, psychomotor speed, and driving simulator operation ability. Although only one study compared the effectiveness of OA with CPAP, the extent of improvement in cognitive function was equivalent in this study.

### 3.4. Psychological disorders

Psychological symptoms, especially depression and anxiety, are commonly reported in individuals with OSA.^[95]^ A community based epidemiological study of 18,980 individuals reported that 17.6% of OSA patients had major depressive disorder, which is very high considering its prevalence of 3% to 4% in the general population.^[96]^ In a meta-analysis, the prevalence of clinically significant depressive symptoms was estimated to be as high as 23% in untreated OSA patients.^[97]^

The relationship between OSA and depression is bidirectional.^[98]^ Sleep deprivation, sleep disturbance, and cognitive changes due to intermittent hypoxemia may cause depression, while weight gain and sleep disturbance due to depression may cause or exacerbate OSA.^[98,99]^ Psychiatric comorbidities are known to adversely affect the QoL of OSA patients and lower adherence to CPAP therapy.^[100,101]^ Whether OSA treatment relieves depressive symptoms is still controversial.^[102]^

Few studies have reported the effect of OA therapy on depressive symptoms. Included studies were based on the Beck Depression Inventory (BDI)^[103]^ and a subscale of the 36-Item Short Form Health Survey (SF-36)^[104]^ to evaluate depressive symptoms. Barnes et al.^[105]^ compared CPAP, MAD, and placebo medication. Clinically significant depression was seen in 40.4% of the OSA patients at baseline. After 4 weeks, BDI scores of all 3 groups decreased, but neither CPAP nor MAD showed a significant improvement beyond a placebo effect. On the other hand, Naismith et al^[90]^ assessed BDI scores and MAD therapy was associated with improvements in somatic items compared with a sham device. Since it is difficult to distinguish certain symptoms of OSA itself from symptoms of depression, the items on sleep quality and daytime hypersomnia included in the BDI questionnaire may confound the interpretation of results. It has been suggested to consider somatic and cognitive components separately when evaluating variables such as mood.^[106]^

Two other studies measuring depressive symptoms with SF-36 did not show significant improvements in SF-36 mental health component scores, despite a significant reduction in AHI after 3 months^[107]^ and 10 weeks^[39]^ of MAD treatment, respectively compared with placebo. Similarly, in a RCT by Petri et al^[108]^ SF-36 mental health component score changed significantly after 4 weeks of MAD treatment, but this difference was not reproduced in covariance analysis. Nevertheless, in a recent meta-analysis including the 5 abovementioned studies, MAD therapy significantly improved depressive symptoms compared with control.^[109]^

Although results are inconclusive due to the small number of studies and their heterogeneity, OA therapy appears to provide minor but significant improvements in depressive symptoms assessed by questionnaires. Further well-designed studies are needed to confirm the effectiveness of OA therapy in ameliorating concomitant depressive symptoms in OSA patients.

### 3.5. Headache

Whether OSA patients experience more headaches than the general population remains controversial, but several studies reported an increased frequency of headaches, especially morning headaches.^[110–113]^ In previous studies, CPAP alleviated sleep-related headaches to some degree.^[114]^

Three studies reporting on the effectiveness of MAD therapy in OSA patients with headaches were identified. Park et al^[115]^ investigated Migraine Disability Assessment (MIDAS) scores and various headache characteristics in 13 headache patients with OSA. After MAD treatment (mean usage duration 5.7 ± 1.1 years), 62% showed a ≥30% reduction in headache frequency. The authors suggested that improvement of intermittent hypoxia through MAD treatment might be insufficient to reduce neurovascular headaches with a stronger genetic component and results may be more favorable with tension-type headaches.^[116]^ The presence of morning headaches was also decreased following MAD treatment. Marklund and Franklin^[117]^ also reported a reduction in self-reported morning headaches after using MAD. Such findings highlight the need to consider OA therapy in treating OSA patients with comorbid headaches and underline the need for routine assessment of headaches during OSA diagnosis. However, in a controlled study,^[118]^ the frequency and intensity of headaches did not differ between MAD and inactive OA treatment. Further studies in patients with specific headache diagnoses are needed to clarify the effect of OA therapy on headaches in OSA patients.

### 3.6. Other measures of systemic health condition

#### 3.6.1. Quality of life.

Daytime symptoms of OSA including excessive daytime sleepiness, lack of concentration, and fatigue negatively affect the QoL of OSA patients.^[119,120]^ The SF-36 was the most widely used health-related QoL questionnaire for OSA patients.^[104]^ In studies comparing the effects of MAD on QoL assessed by SF-36 with inactive devices, Petri et al^[108]^ and Marklund et al^[118]^ found no significant difference in the extent of improvement in QoL between the 2 groups. In studies comparing CPAP and MAD, both treatments resulted in similar levels of improvement in QoL.^[94,121]^ Even in veterans with posttraumatic stress disorder, MAD therapy was equally effective as CPAP in reducing PTSD severity and improving health-related QoL.^[121]^ In 2 recent meta-analyses comparing the effectiveness of CPAP and MAD, both treatments showed similar extents of improvements with SF-36.^[122,123]^ However, in the meta-analysis by Kuhn et al,^[122]^ the evidence for MAD was weaker than for CPAP to support the improvement in QoL due to the relatively small amount of data related to MAD. Health-related QoL is one of the most important health outcomes of OSA intervention for patient-centered care. Hence further evidence is needed from large scale RCTs to investigate the true impact of OA therapy on QoL.

#### 3.6.2. Telomere length.

Telomeres are complexes of repetitive DNA sequences and proteins that protect the end of the chromosome from degradation and fusion.^[124]^ In humans, telomere shortening that occurs throughout the lifespan is associated with cancer,^[125]^ cardiovascular diseases,^[126]^ and increased mortality.^[127]^ Another molecule, Sirtuin 1 (SIRT1), regulates various signaling pathways involved in metabolism, inflammation, cellular senescence, proliferation, apoptosis, and DNA damage. SIRT1 is known to play an important role in age-related diseases.^[128,129]^ Hypoxia, oxidative stress, and systemic inflammation accelerate the shortening of telomere and disrupt SIRT1 activity.^[130]^

Previous studies have shown that leukocyte telomere length (LTL) is shorter^[131]^ and SIRT1 protein levels are lower in OSA patients compared with healthy subjects.^[132]^ A few studies reported recovery in blood levels of SIRT1 protein following successful nasal CPAP treatment of OSA.^[132]^ One clinical trial which investigated the effect of MAD treatment on circulating LTL and SIRT1 protein levels in peripheral blood mononuclear cells with 40 moderate to severe OSA patients for 3 months reported that OSA patients had lower LTL and SIRT1 protein levels compared with healthy subjects. After 3 months of MAD treatment, LTL and SIRT1 protein levels were normalized in 24 MAD responders (defined as post-treatment AHI <5). However, LTL and SIRT1 protein levels remained low in the 16 MAD non-responders.^[133]^

Short LTL and low SIRT1 levels reflect accelerated cellular senescence in OSA patients and such abnormalities would increase the risk of age-related diseases.^[134]^ Although further studies are needed, the data until now shows that successful OSA treatment effectively restores telomere length and blood levels of the SIRT1 protein. LTL and SIRT1 could be used as markers to confirm the effectiveness of the OSA intervention including OA.

## 4. Conclusions

The results accumulated until now on the systemic effects of OA therapy support its widespread application for OSA patients of diverse severities. Among the various systemic effects investigated in relation to OA therapy, cardiovascular outcomes, particularly blood pressure was the most studied suggesting that OAs may have a small but significant beneficial effect on high blood pressure. OA therapy also showed positive effects on metabolic consequences, cognitive function, depressive symptoms, headache, quality of life, and telomere length. However, such conclusions are limited due to the small number of related studies, small sample size of individual studies, and heterogeneity in study design. Further evidence from well-designed large-scale RCTs on a wider range of health conditions is needed to further elucidate the systemic impact of OA therapy in OSA patients. Future studies should apply objective compliance measurement for more accurate results.

## Author contributions

Conceptualization: Ji Woon Park.

Data curation: Hee Young Kim.

Formal analysis: Hee Young Kim, Ji Woon Park.

Investigation: Hee Young Kim, Ji Woon Park.

Methodology: Jung Hwan Jo, Jin Woo Chung, Ji Woon Park.

Project administration: Ji Woon Park.

Resources: Jung Hwan Jo, Ji Woon Park.

Software: Hee Young Kim.

Supervision: Ji Woon Park.

Validation: Jung Hwan Jo, Jin Woo Chung.

Visualization: Hee Young Kim.

Writing – original draft: Hee Young Kim, Ji Woon Park.

Writing – review & editing: Jung Hwan Jo, Jin Woo Chung, Ji Woon Park.

Conceptualization: Ji Woon Park

Data curation: Hee Young Kim

Formal analysis: Hee Young Kim, Ji Woon Park

Investigation: Hee Young Kim, Ji Woon Park

Methodology: Ji Woon Park, Jin Woo Chung, Jung Hwan Jo

Project administration: Ji Woon Park

Resources: Ji Woon Park, Jung Hwan Jo

Software: Hee Young Kim

Supervision: Ji Woon Park

Validation: Jin Woo Chung, Jung Hwan Jo

Visualization: Hee Young Kim

Writing – original draft: Hee Young Kim

Writing – review & editing: Ji Woon Park, Jin Woo Chung, Jung Hwan Jo
